# *BDNF* Val66Met Polymorphism and Gamma Band Disruption in Resting State Brain Functional Connectivity: A Magnetoencephalography Study in Cognitively Intact Older Females

**DOI:** 10.3389/fnins.2018.00684

**Published:** 2018-10-02

**Authors:** Inmaculada C. Rodríguez-Rojo, Pablo Cuesta, María Eugenia López, Jaisalmer de Frutos-Lucas, Ricardo Bruña, Ernesto Pereda, Ana Barabash, Pedro Montejo, Mercedes Montenegro-Peña, Alberto Marcos, Ramón López-Higes, Alberto Fernández, Fernando Maestú

**Affiliations:** ^1^Laboratory of Cognitive and Computational Neuroscience, Center for Biomedical Technology, Universidad Complutense and Universidad Politécnica de Madrid, Madrid, Spain; ^2^Department of Experimental Psychology, Cognitive Processes and Speech Therapy, Universidad Complutense de Madrid, Madrid, Spain; ^3^Electrical Engineering and Bioengineering Lab, Department of Industrial Engineering and IUNE, Universidad de La Laguna, Tenerife, Spain; ^4^Biological and Health Psychology Department, Universidad Autónoma de Madrid, Madrid, Spain; ^5^Networking Research Center on Bioengineering, Biomaterials and Nanomedicine (CIBER-BBN), Madrid, Spain; ^6^Laboratory of Psychoneuroendocrinology and Genetics, Hospital Clínico San Carlos, Madrid, Spain; ^7^Instituto de Investigación Sanitaria del Hospital Clínico San Carlos (IdISSC), Madrid, Spain; ^8^Center for the Prevention of Cognitive Impairment, Public Health Institute, Madrid-Salud, Madrid, Spain; ^9^Neurology Department, Hospital Clínico San Carlos, Madrid, Spain; ^10^Department of Legal Medicine, Psychiatry, and Pathology, Universidad Complutense de Madrid, Madrid, Spain

**Keywords:** *BDNF* Val66Met, magnetoencephalography, gamma rhythms, cognitive functioning, healthy aging, Alzheimer's disease

## Abstract

The pathophysiological processes undermining brain functioning decades before the onset of the clinical symptoms associated with dementia are still not well understood. Several heritability studies have reported that the Brain Derived Neurotrophic Factor (*BDNF*) Val66Met genetic polymorphism could contribute to the acceleration of cognitive decline in aging. This mutation may affect brain functional connectivity (FC), especially in those who are carriers of the *BDNF* Met allele. The aim of this work was to explore the influence of the *BDNF* Val66Met polymorphism in whole brain eyes-closed, resting-state magnetoencephalography (MEG) FC in a sample of 36 cognitively intact (CI) older females. All of them were ε3ε3 homozygotes for the apolipoprotein E (*APOE*) gene and were divided into two subgroups according to the presence of the Met allele: Val/Met group (*n* = 16) and Val/Val group (*n* = 20). They did not differ in age, years of education, Mini-Mental State Examination scores, or normalized hippocampal volumes. Our results showed reduced antero-posterior gamma band FC within the Val/Met genetic risk group, which may be caused by a GABAergic network impairment. Despite the lack of cognitive decline, these results might suggest a selective brain network vulnerability due to the carriage of the *BDNF* Met allele, which is linked to a potential progression to dementia. This neurophysiological signature, as tracked with MEG FC, indicates that age-related brain functioning changes could be mediated by the influence of particular genetic risk factors.

## Introduction

Over the last two decades, the interest in exploring candidate genes related to cognitive and brain aging has significantly increased (Kennedy et al., 2014). The Brain Derived Neurotrophic Factor (*BDNF*) is one of the genes involved in the aging process due to its protective role in neural function (Chen et al., 2013; Rothman and Mattson, 2013; Marosi and Mattson, 2014; Criscuolo et al., 2015). The molecule derived from the BDNF expression is one of the most abundant brain neurotrophins (Zhang et al., 2007), and its presence is essential for the maintenance, survival, and growth of neurons (Cohen-Cory and Fraser, 1995; Mattson et al., 2004). BDNF is also related to the activity-dependent synaptic plasticity, which is crucial for the modulation of several cognitive domains such as learning, memory, and executive functions (Horch et al., 1999; Egan et al., 2003; Binder and Scharfman, 2004; Bath and Lee, 2006). However, it is well-known that BDNF production decreases with age (Mattson and Magnus, 2006) and more importantly, with the emergence of Alzheimer's Disease (AD)-related neuropathology (Qin et al., 2017). With this background in mind, recent literature (Matyi et al., 2017) has focused on exploring the association between several *BDNF* single nucleotide polymorphisms (SNPs) and this neurodegenerative disorder, providing additional evidence in support of the interest of further studying this relationship.

Of particular relevance is the role of the Val66Met *BDNF* SNP, where a Valine (Val) is substituted by a Methionine (Met) at codon 66 (Val66Met, rs6265, G>A) on chromosome 11 (Egan et al., 2003). This functional mutation alters the intracellular trafficking and packaging of proBDNF, affecting the regular activity-dependent expression of the mature BDNF peptide (Egan et al., 2003; Chen et al., 2004). Specifically, Met allele carriers (Val/Met) exhibit a lower BDNF secretion than Val/Val homozygotes (Val/Val) (Kennedy et al., 2014). This alteration has been related to increased hippocampal atrophy (Lim et al., 2013, 2014a; Rabl et al., 2014), loss of white matter integrity (Kennedy and Raz, 2009; Voineskos et al., 2011; Ziegler et al., 2013), greater cognitive decline or higher susceptibility to AD (Voineskos et al., 2011; Kambeitz et al., 2012; Huang et al., 2014; Lim et al., 2014a), and poorer neurocognitive performance in healthy controls (Dincheva et al., 2012).

In addition to genetic approach, the detection of abnormal changes in the brain's oscillatory activity is considered nowadays as one of the most suitable strategies in the study of neurodegenerative disorders (Pievani et al., 2011; Başar and Düzgün, 2016). Functional connectivity (FC) estimators are good examples of such new strategies, since they enable the investigation of the brain from the perspective of a complex neural network. As a consequence, brain diseases are contemplated as network disruption processes (Varela et al., 2001; Stam, 2014). Electroencephalography (EEG) and magnetoencephalography (MEG) are non-invasive neuroimaging techniques that directly measure primary neural activity, and provide useful markers to characterize functional synaptic alterations (Stam, 2010; López et al., 2014; Pievani et al., 2014; Nakamura et al., 2017). *BDNF* has a regulatory role in the synaptic function, and several studies have reported that the Met allele contributes to the alteration of the morphology and FC in the brain (Beste et al., 2010; Jang et al., 2012; Wei et al., 2012). Therefore, MEG FC emerges as an appropriate tool to assess the effects of *BDNF* in Met carriers and non-carriers.

Considering the aforementioned, we hypothesize that the carriage of a Met allele (i.e., a lower secretion of BDNF) would produce a disruption in the transmission of brain signals that, in turn, alters FC concomitantly to a poorer cognitive performance. To the best of our knowledge, this is the first study assessing the effect of the *BDNF* Val66Met SNP in MEG functional brain networks. The multimodal analysis undertaken to test this hypothesis also involved structural (i.e., medial temporal lobe volumes), cognitive (i.e., neuropsychological tests), and *APOE* genotyping information from a group of cognitively intact (CI) older females due to their suggested higher predisposition to AD's (Barnes et al., 2005; Fleisher et al., 2005; Neu et al., 2017; Fisher et al., 2018).

## Materials and methods

### Participants

The original sample consisted of 36 CI older Caucasian females recruited from the “Hospital Universitario San Carlos”, and the “Center for the Prevention of Cognitive Impairment” of Madrid. All participants underwent an extensive neuropsychological assessment to explore their cognitive functioning, which included the following tests: the Spanish version of the Mini-mental state Examination (MMSE, Lobo et al., 1979), forward and backward digit spam tests (FDS and BDS, Wechsler Memory Scale III, WMS-III; Wechsler, 1997), immediate and delayed recall (IR and DR, WMS-III; Wechsler, 1997), phonemic and semantic fluency (PhF and SF, controlled oral word association test; Benton and Hamsher, 1989), Boston naming test (BNT, Kaplan et al., 1983), and trail-making test (TMT) parts A and B (TMT-A and TMT-B, Reitan, 1958). They also underwent a multimodal neuroimaging assessment including Magnetic Resonance Imaging (MRI) and MEG scans. All of them were right-handed, native Spanish speakers and did not differ in age, years of education, MMSE (Folstein et al., 1975; Lobo et al., 1979) score, or normalized hippocampal volumes (see Table 1 for information relative to the characteristics of the participants).

**Table 1:** Characteristics of the participants.

	**Val/Val** **(*n* = 20)**	**Val/Met** **(*n* = 16)**	**Statistics** **(*p* value)**
Age	70.1 ± 4.8	70.3 ± 4.9	0.810
Education (years)	12.6 ± 4.2	11.3 ± 5.3	0.453
MMSE	29.3 ± 1.0	29.5 ± 0.7	0.505
BNT	54.0 ± 4.3	51.4 ± 8.4	0.238
Forward digit span	8.7 ± 2.6	8.0 ± 1.5	0.345
Backward digit span	6.3 ± 2.1	5.1 ± 1.7	0.066
Immediate recall	39.8 ± 9.9	39.9 ± 20.1	0.970
Delayed recall	26.4 ± 7.3	25.6 ± 10.0	0.802
Phonemic fluency	14.6 ± 3.8	13.9 ± 4.7	0.656
Semantic fluency	16.8 ± 3.8	17.4 ± 5.0	0.647
TMT-A (time)	48.4 ± 13.4	58.3 ± 24.0	0.125
TMT-B (time)	107.6 ± 46.6	138.1 ± 77.0	0.144
Left hippocampal volume	0.0027 ± 0.0003	0.0027 ± 0.0004	0.987
Right hippocampal volume	0.0028 ± 0.0003	0.0027 ± 0.0004	0.729
Left entorhinal volume	0.0016 ± 0.0002	0.0016 ± 0.0003	0.854
Right entorhinal volume	0.0017 ± 0.0003	0.0016 ± 0.0003	0.232

We present values as mean ± SD. Statistical analyses were performed using one-way ANCOVA (age as confounding covariate).

MMSE, Mini-Mental State Examination; TMT-A, Trail-Making Test part A; TMT-B, Trail-Making Test part B; BNT, Boston Naming Test.

*Medial temporal lobe volumes were normalized with respect to the overall intracranial volume to account for differences in head volume over subjects*.

Individuals with any significant medical, neurologic or psychiatric diseases were excluded from the study according to their medical history, neurologic and clinical examination, and MRI results. Inclusion criteria were the absence of significant cerebral-vascular disease (modified Hachinski score ≤ 4) and depressive symptomatology (Yesavage's Depression Scale scores > 9), and an age between 60 and 80 years. In addition, T2-weighted MRIs within 12 months before MEG screening should not show any indication of infection, infarction, or focal lesions (rated by two independent experienced radiologists (Bai et al., 2012).

Concerning the genetic load, participant's selection was restricted to those homozygous for the *APOE* ε3 allele. Different studies have shown the deleterious effect produced by the combination of being carrier of both the *BDNF* Met allele and the *APOE* ε4 allele (Hashimoto et al., 2009; Adamczuk et al., 2013; Kauppi et al., 2014; Lim et al., 2014b). Based on this, we selected only *APOE* ε3ε3 individuals, who in turn were classified as carriers and non-carriers of the *BDNF* Met allele (i.e., Val/Met vs. Val/Val), since Met/Met homozygotes were not sufficiently prevalent to conduct specific subgroup analysis.

The Hospital Universitario San Carlos local Ethics Committee approved the study, and all the participants provided written informed consent in accordance with the Declaration of Helsinki and prior to their examination to participate in the clinical studies of cognitive impairment.

### *BDNF* and *APOE* genotype test

DNA was extracted from whole-blood samples of CI older females. As previously described in (Cuesta et al., 2015), *APOE* haplotype was determined by analyzing SNPs rs7412 and rs429358 genotypes with TaqMan assays using an Applied Biosystems 7900 HT Fast Real Time PCR machine (Applied Biosystems, Foster City, CA). The same procedure was applied to analyze the *BDNF* rs6265 (Val66Met) SNP. A genotyping call rate over 90% per plate, sample controls for each genotype and negative sample controls were included in each assay. Three well-differentiated genotyping clusters for each SNP were required to validate results. Intra and inter-plate duplicates of several DNA samples were included.

### Structural MRI

We collected 3D T1 weighted anatomical brain MRI scans with a General Electric 1.5T MRI scanner, using a high-resolution antenna and a homogenization PURE filter (Fast Spoiled Gradient Echo (FSPGR) sequence with parameters: TR/TE/TI = 11.2/4.2/450 ms; flip angle 12°; 1 mm slice thickness, a 256 × 256 matrix and FOV 25 cm). The MRIs were processed with Freesurfer software (version 5.1.0.21) to obtain the volume of gray matter in several brain areas (Fischl et al., 2002), which were normalized with respect to the overall intracranial volume (ICV).

### MEG

#### Acquisition

MEG signals were acquired using a whole-head Elekta-Neuromag MEG system with 306 channels (Elekta AB, Stockholm, Sweden), placed in a magnetically shielded room (VacuumSchmelze GmbH, Hanua, Germany), at the Center for Biomedical Technology (Madrid, Spain). Participants sat comfortably on a chair with their eyes closed while 4 min of resting state MEG signals data were collected with a sampling frequency of 1,000 Hz and online band-pass filtered between 0.1 and 330 Hz. The positions of four head-position indicator (HPI) coils attached to the scalp, and each subject's head shape relative to three anatomical locations (nasion and both preauricular points) were defined using a 3D digitizer (Fastrak, Polhemus, VT, USA). The HPI coils continuously controlled the participants' head movements, whereas eye movements were monitored by means of a vertical electrooculograph using a pair of bipolar electrodes. Finally, we supervised the arousal level of each subject through a video camera and checked it via a conversation immediately after the measurement session.

#### Preprocessing

In order to remove environmental noise, MEG raw data were first submitted to the Maxfilter software with the temporal extension of the signal space separation method (v 2.2, correlation threshold = 0.9, time window = 10 s) with movement compensation (Taulu and Simola, 2006). MEG data were automatically scanned for ocular, muscle, and jump artifacts using the Fieldtrip software (Oostenveld et al., 2011). Afterwards, artifacts were visually confirmed by a MEG expert. We segmented the remaining artifact-free data in 4-s segments (trials) and used then in an independent component analysis-based procedure to remove the heart artifact. At least 20 clean 4-s-long trials (80 s of brain activity) were then obtained from each participant, and 20 of these clean trials were randomly selected from each subject to equalize the number of trials. Previously to source data calculation, MEG time series were filtered into theta (4–8 Hz), alpha (8–12 Hz), beta (12–30 Hz), and gamma (30–45 Hz) frequency bands with a 1,500 order finite impulse response filter designed using Hamming window and a two-pass filtering procedure.

#### Source reconstruction

A regular volumetric grid with 10 mm spacing was created in MNI space. This set of nodes was transformed to each participant's space using a non-linear normalization between the native T1 image (whose coordinate system was previously converted to match the MEG coordinate system) and a standard T1 in MNI space. The forward model was solved using the realistic single-shell model introduced by Nolte (2003). Sources reconstruction was performed independently for each subject and frequency band, using a linearly constrained minimum variance (LCMV) beamformer (Van Veen et al., 1997). In order to combine the two types of sensors in the MEG scan, the leadfields were normalized by sensor type, and the same scaling factor was applied to the data. Beamformer filters were estimated from the average covariance of the trials using a 15% regularization factor. The neural MEG source-space time series were anatomically parcellated by dividing the cortex into 90 regions of interest (ROIs) according to the AAL atlas (Tzourio-Mazoyer et al., 2002). After excluding the cerebellum, basal ganglia, thalamus, amygdala, insula and olfactory cortices, the anatomical model consisted of 72 ROIs. Per each one of them, we selected the first component, obtained with a principal component analysis (PCA), as the representative time series. Finally, the FC was assessed using the phase locking value (PLV), a phase synchronization measure that evaluates the distribution of phase differences extracted from each two ROIs time series (Mormann et al., 2000).

### Statistical analysis

The procedure for statistical comparisons relied on the network based statistics (NBS) introduced by Fornito et al. (2016) and Zalesky et al. (2010), which was carried out independently for each frequency band. The aim of this piece of work was the detection of any robust significant connected subnetworks (assembly of nodes for which a path can be found linking any pair of nodes in that subnetwork). These subnetworks consisted of several significant links (*p*-value of 0.005, ANCOVA test with age as covariate), which systematically should show either diminished or enhanced FC in the Val/Met group compared with the Val/Val homozygotes. Network-statistics was assessed through the sum of all F-values corresponding with the subnetwork's links. Candidate subnetworks were required to have a minimum size defined by the obligation of involving at least the 10% of the ROIs of the model (i.e., 7 ROIs for our atlas model). Then, to control for the multiple comparisons problem, the entire analysis pipeline was repeated 5,000 times after shuffling the original group's labels. At each repetition, the maximum statistic of the surrogate subnetworks was kept creating a maximal null distribution that ensured control of the family-wise error rate (FWER) at the subnetwork level. The network-statistics over each subnetwork in the original data set, was compared with the same measure in the randomized data. The NBS *p*-value represented the proportion of the permutation distribution with network-statistic values greater or equal than the network-statistic value of the original data. Only those subnetworks that survived after the NBS were used for the subsequent analyses as potential “MEG markers.” As descriptive values for each significant subnetwork, we computed their average FC (across all links that belong to the subnetwork, i.e., their corresponding strength) values. These data were then used in the exploratory analysis for the correlation (Spearman's correlation) with the neuropsychological scores. In addition, for these strength values, we computed between-group statistical analysis using an ANCOVA with age as covariate. Results were not corrected for the multiple comparisons associated with the assessment of between-group differences in functional brain network for 5 frequency bands. Finally, to rank network's links based on their accuracy to distinguish between both groups, a logistic regression classification analysis with a leave-one-out cross-validation procedure was performed assessing the subnetwork-representative strength and the FC values of the individual links (see López et al., 2014). For classification purposes, Val/Met group was considered deleterious. Accuracy value represents the fraction of participants correctly classified. Sensitivity and specificity values represent the fractions of Val/Met and Val/Val individuals correctly classified, respectively. Finally, positive predictive values (PPVs) and negative predictive values (NPVs) represent the fraction of individuals classified as Val/Met that were really Val/Val and the fraction of participants classified as Val/Val that were really Val/Met. Statistical analyses were carried out using Matlab R2016a (Mathworks Inc).

## Results

### Participant data

Table 1 shows the characteristics of the participants. There were no significant (p > 0.05) differences in age, educational attainment, neuropsychological performance or hippocampal/entorhinal volumes (Table 1).

**Figure 1:**
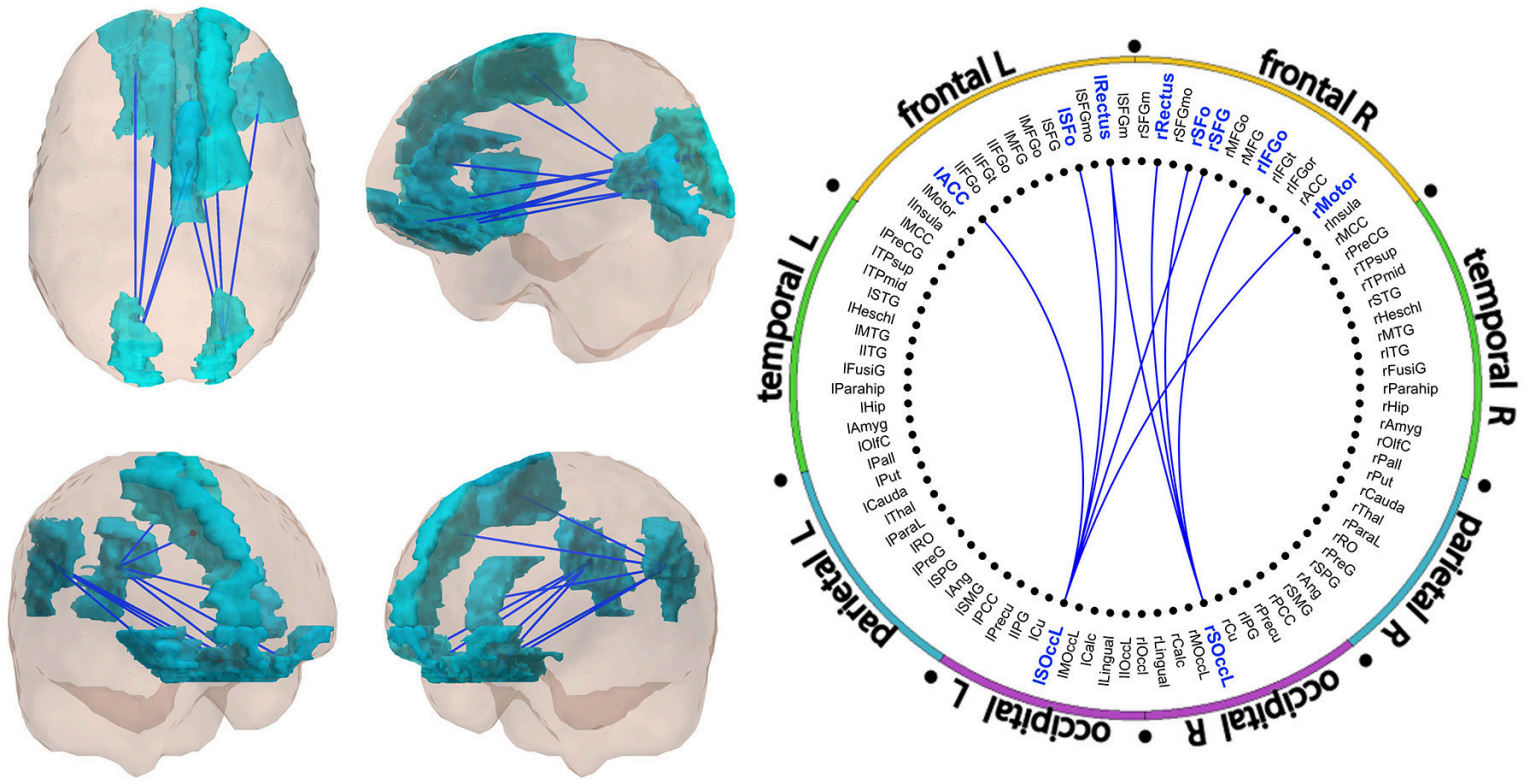
Significant subnetwork in the gamma band. All links showed decreased FC in Val/Met individuals as compared to Val/Val participants. A schematic representation of the links is showed in Figure 2. FC values of the links are described in Table 2.

**Figure 2:**
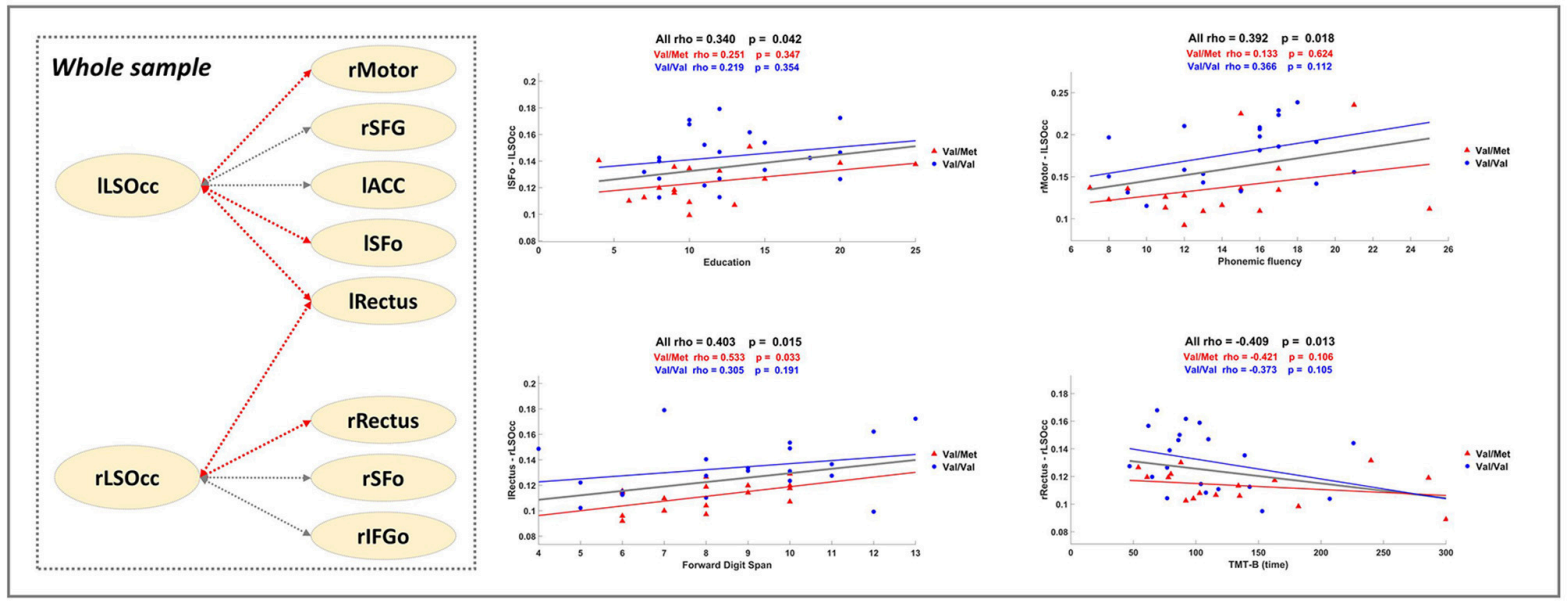
Correlation Analysis. The dashed square in the left part presents a summary of the significant results described in Table 3. Gray dashed lines correspond to the links of the significant subnetwork. Red dashed lines indicate that the FC of the link is positively correlated with better performance. Scatter plots show some significant Spearman's rho values computed with all individuals within the sample (see bold highlighted values in Table 3). Blue and red lines depict, respectively, extra regression analysis carried out uniquely with Val/Val and Val/Met groups (for information purposes).

Results of the FC analysis are summarized in Figures 1, 2, and Table 2. The network-based FC methodology pointed out the existence of an altered brain network composed by long-range connections between antero-posterior ROIs, showing a significant diminished FC in Val/Met participants when compared to Val/Val homozygotes. This alteration was restricted to the gamma frequency band.

**Table 2:** Functional connectivity results.

	**FC Val/Val**	**FC Val/Met**	**Statistics (*p*-value)**	**Classification**						
				**Accuracy**	**Sens**	**Spec**	**PPV**	**NPV**	**Upper B**	**Lower B**
Subnetwork strength	0.149 ± 0.015	0.123 ± 0.010	0.000001	**0.861**	**0.875**	**0.850**	**0.824**	**0.895**	**0.953**	**0.705**
lLSOcc–rSFG	0.159 ± 0.034	0.129 ± 0.020	0.004026	0.722	0.750	0.700	0.667	0.778	0.858	0.548
lLSOcc–lSFo	0.143 ± 0.020	0.124 ± 0.015	0.003335	0.611	0.625	0.600	0.556	0.667	0.769	0.435
lLSOcc–rMotor	0.178 ± 0.036	0.137 ± 0.040	0.003255	0.694	0.813	0.600	0.619	0.800	0.837	0.519
lLSOcc–lRectus	0.153 ± 0.031	0.124 ± 0.019	0.002542	0.667	0.688	0.650	0.611	0.722	0.814	0.490
lLSOcc–lACC	0.186 ± 0.041	0.145 ± 0.036	0.003522	0.694	0.813	0.600	0.619	0.800	0.837	0.519
rLSOcc–rSFo	0.129 ± 0.021	0.110 ± 0.011	0.001246	0.722	0.813	0.650	0.650	0.813	0.858	0.548
rLSOcc–rIFGo	0.127 ± 0.019	0.111 ± 0.007	0.003521	0.750	0.875	0.650	0.667	0.867	0.879	0.578
rLSOcc–lRectus	0.134 ± 0.022	0.111 ± 0.011	0.000651	0.806	0.875	0.750	0.737	0.882	0.918	0.640
rLSOcc–rRectus	0.131 ± 0.022	0.113 ± 0.012	0.003927	0.667	0.750	0.600	0.600	0.750	0.814	0.490

Functional Connectivity (FC) values are presented as mean ± SD. Statistical analyses were performed using one-way ANCOVA (age as confounding covariate). The accuracy, sensitivity and specificity, negative predictive value (NPV) and positive predictive value (PPV) scores were obtained through a logistic regression analysis with leave-one-out cross-validation procedure. The confidence interval (Upper and Lower B) (CI) for the statistic, was calculated using the β approach (**in bold**, the highest scores of each variable).

*l/r-LSOcc, left/right lateral superior occipital lobe; rMotor, right supplementary motor area; rSFG, right superior frontal gyrus; l/r-Rectus, left/right gyrus rectus; l/r-SFo, left/right orbital superior frontal gyrus; lACC, left anterior cingulate gyrus; rIFGo, right orbital inferior frontal gyrus*.

Gamma subnetwork (CBPT *p* = 0.041) comprised nine significant links (Figures 1, 2 and Table 2), with a clear antero-posterior pattern in which both (left and right) lateral superior occipital ROIs (l- and rLSOcc) connected with different frontal ROIs. The pattern of connections of the subnetwork (Figure 2) showed that both LSOcc ROIs played a role as hubs in two different sub-networks, uniquely connected to each other through the left gyrus rectus ROI. The lLSOcc connected with the right supplementary motor area (rMotor), the right superior frontal gyrus (rSFG), the left gyrus rectus (lRectus), the left orbital superior frontal gyrus (lSFo), and the left anterior cingulate cortex (lACC). On the other hand, the rLSOcc connected with the right orbital inferior frontal gyrus (rIFGo), the right orbital superior frontal gyrus (rSFo) and both gyrus rectus.

### Correlations analysis between MEG FC data and neuropsychological assessment

To further elucidate the relevance of the MEG FC markers to assess cognitive performance, *a set of exploratory* correlation analyses between the MEG FC marker values and cognitive scores were performed (Figure 2 and Table 3). Figure 2 displays a schematic representation of the significant subnetwork, where we have depicted the relationship between links and the results of the correlation analysis, which was carried out including the whole sample and showed a positive correlation between FC values and better cognitive performance (FDS, BDS PhF) and education. Age was negatively associated with the FC between rRectus and rLSOcc ROIs in the Val/Val group. A similar correlation was found in the Val/Met group for the FC between lRectus and rLSOcc, although in this case the *p*-value was slightly above the threshold (*p* < 0.07).

**Table 3:** FC markers correlation analysis.

	**lLSOcc–lSFo**	**lLSOcc–rMotor**	**lLSOcc–lRectus**	**rLSOcc–lRectus**	**rLSOcc–rRectus**
Age				(−0.48 ^*^)	[−0.51]
Education	**0.34**				
Forward digit Span				**0.40 (0.53)**	
Backward digit Span			0.33	0.33	0.34
Phonemic fluency		**0.39**	[0.50]		
TMT-B (time)					–**0.41**

Correlation analysis (Spearman rho values significant at p < 0.05) are shown for each FC marker. Rho values are presented on basis of the sample used in the analysis: All sample, (Val/Met sample) or [Val/Val sample].

TMT-B, Trail-Making Test part B; l/r-SOccL, left/right superior occipital lobe; rMotor, right Supplementary motor area; rSFG, right superior frontal gyrus; l/r-Rectus, left/right gyrus rectus; l/r-SFo, left/right orbital superior frontal gyrus.

Significant correlations are depicted in Figure 2.

* *Result with 0.05 < p < 0.06*.

*Bold highlighted results are shown in Figure 2*.

### Classification

In order to rank the FC results based on their capability to distinguish between both groups, a classification analysis was performed independently per every individual FC value found in the study, including links and subnetwork strength. The highest accuracy (86.1%) was obtained for the subnetwork strength value, followed by the result of the lRectus–rLSOcc link (80.6%), which supports our approach of focusing on networks rather than individual links. All the classification results are shown in Table 2.

## Discussion

In this research, we have carried out a MEG FC analysis to examine the influence of *BDNF* Val66Met SNP in CI older females. We found that Met allele carriers showed a diminished antero-posterior gamma band FC, which might be caused by a reduction in the ability to synchronize corresponding brain areas as accurately as Val/Val participants (Beste et al., 2010). It has been reported that gamma oscillations mainly emerge from the coordinated interaction between excitatory-inhibitory neuronal activity (Buzsáki and Wang, 2012). In fact, GABAergic interneurons (i.e., parvalbumin-positive interneurons) are the main generators of gamma oscillations (Başar, 2013). *In silico* models of GABAergic network impairment (e.g., reduced GABAergic innervation or connectivity, or a long decay time of the inhibitory activity), together with studies conducted in AD patients, evidenced a dramatic reduction in the gamma band activity in this population (Stam et al., 2002; Başar, 2013; Başar et al., 2016; Metzner et al., 2016). On top of that, BDNF has also been found to influence GABAergic interneurons (Holm et al., 2009), and, as a synaptic trophic factor strengthening connections between brain networks, modulates gamma oscillations (Tamura et al., 2017). Therefore, the Met allele carriage apparently weakens this excitatory-inhibitory connection by reducing the amplitude and synchronization of gamma brain waves. Furthermore, the observed FC disruption involves several long-range connections (e.g., the lRectus-rLSOcc with an 80.6% of classification), demonstrating that synchronization in this frequency band has a role not only in local, but also in long-distance networks (Başar, 2013).

Similarly, it should be noted that the alteration of gamma oscillations in resting-state within the AD continuum is still controversial and little explored. Different studies have reported conflicting results showing increased or decreased gamma band activity (Stam et al., 2002; Stam, 2010; Wang et al., 2017). These discrepancies may be due, among other factors, to the severity of the disease and the experimental conditions applied (i.e., task vs. resting-state) (Wang et al., 2017). For example, gamma activity (both spontaneous or evoked/induced), has been located in many cortical brain areas (Fries, 2009) being involved in multiple cognitive functions (Başar, 2013; Başar et al., 2015). In addition, Buzsáki and Wang (Buzsáki and Wang, 2012) have stated that the activation of this oscillatory rhythm could vary depending on the underlying mechanism considered. On the other hand, the Met allele mutation in the 5′ pro-region of the BDNF protein (possibly leading to an excessive proBDNF production which could be toxic to neurons), produces an imbalance in the correct release of the mature BDNF peptide (Egan et al., 2003; Chen et al., 2004). Besides, BDNF expression is equitably ubiquitous throughout the brain (Zhang et al., 2007), and its receptors are abundantly expressed in parvalbumin-positive interneurons, which are selectively regulated by binding to the mature form of BDNF (Holm et al., 2009). As previously said, these GABAergic interneurons are crucial for the emergence of the gamma band oscillatory response (Zheng et al., 2011 as cited by Tamura et al., 2017). Thus, it may be that due to this functional mutation, the proBDNF is not only unable to adequately modulate GABAergic activity (Holm et al., 2009), but also to inhibit BDNF's availability, contributing to a potential neurobiological loss of function (Uegaki et al., 2017). All these evidences support the hypo-synchronized gamma band pattern found in the Met carrier group, highlighting that *BDNF* Val66Met SNP may exert an influence on brain connectivity in healthy and pathological aging.

Regarding the relationship between significant FC results and neuropsychological scores, correlation analyses indicated that enhanced FC in the gamma subnetwork was associated with better cognitive performance (i.e., FDS, BDS and PhF) in the whole sample, but, as can be observed in the regression scatter plots (Figure 2), Val/Val homozygotes showed on average more FC. In line with this result, we would like to underscore the positive correlation between the lSFo-lLSOcc FC and education. Education is a well-established proxy of cognitive reserve (CR) (Stern, 2012), and a recent study in CI older adults across a 36-month follow-up period concluded that CR-related differences in executive function decreased in both Val/Val and Val/Met participants, but became more notable in Met carriers (Ward et al., 2017). In our study, both groups showed a similar rho value when the correlation between lSFo-lLSOcc FC and education was performed for each group separately (Figure 2). However, the fact that the two groups behaved similarly and given the positive correlations between FC and cognitive performance, it could be suggested that the antero-posterior network activity differences found in our sample could be interpreted as a signature of the underlying mechanisms involved in CR. Following Ward et al. (2017) findings, we would expect that the strength of the correlation between FC and the cognitive status will deteriorate to a larger extent for Val/Met participants, constituting a downstream marker of aging-related neurodegeneration. Finally, the negative association between the rRectus-rLSOcc FC and age within the Val/Val group (not achieving statistical significance for the Val/Mets) supports the evidence that BDNF levels decrease throughout the aging process (Mattson and Magnus, 2006; Sohrabji and Lewis, 2006) and, therefore, another possible mechanism of greater susceptibility to age-related cognitive impairment.

From our point of view, these results suggest that electrophysiological resting-state FC, as measured by MEG, could be potentially considered as a non-invasive, sensitive biomarker enabling the assessment of brain functional integrity associated with genetic factors (Schofield et al., 2009; Voineskos et al., 2011; Papenberg et al., 2015; Chiesa et al., 2017), such as the *BDNF* Val66Met SNP. FC studies like the present one are expected to deepen the understanding of the pathophysiological processes underlying healthy and pathological aging (Babiloni et al., 2016; Walsh et al., 2017).

## Limitations and future approaches

The evidence presented here emphasizes the relevance of the neuroimaging genetics field. However, there are several methodological issues that should be considered and that, as in many other cases could account for the conflicting results found through the literature on *BDNF* Val66Met SNP (for more information see Notaras and Hill, 2015). For example, the effect of the Met/Met genotype could not be appropriately examined (Zdanys et al., 2009; Forde et al., 2014). This is an important issue because the reduced dependent release of BDNF could vary from 18% for one Met allele to 29% in the Met/Met homozygous cases (Chen et al., 2006). In addition, analyzing additive or gene-gene interactions (Kauppi et al., 2014; Kennedy et al., 2014), ethnicity (Brooks et al., 2014; Lin et al., 2014), other BDNF's SNPs (e.g., rs56164415 or rs2072446) (Matyi et al., 2017), their epigenetic modulation, and gender differences, could also contribute to clarify BDNF's role in human brain functioning and neurodegeneration (Boulle et al., 2012; Honea et al., 2013).

Besides, we only selected CI older females who were *APOE* ε3ε3, not including *APOE* ε4 allele carriers, neither its interaction with *BDNF* Val66Met SNP. Anyway, a study conducted by Sen and co-workers found that *APOE* ε3 had little or no effect on BDNF levels (Sen et al., 2015 as cited by Fisher et al., 2018). This supports, on the other hand, the suitability of the selected study sample, since our results could be more purely restricted to the *BDNF* Val66Met SNP effects. In addition, while several studies have reached the conclusion that age-related pathological changes in brain structure and function could occur independently of genetic influence (Erickson et al., 2012), others claim that the genetic harmful effects may emerge as a factor of age (i.e., the antagonistic pleiotropy theory of aging) (Williams, 1957; Li et al., 2010; Voineskos et al., 2011). Therefore, the implementation of longitudinal studies could be an interesting way to shed light on how different genetic variations could affect different brain disorders throughout the entire life cycle (Gruber et al., 2012; Papenberg et al., 2015).

Finally, three more concerns should be highlighted. The first one is related to the non-existence of hippocampal volume differences between groups as reported in Lim et al. (2013, 2014a) and Rabl et al. (2014). We would like to clarify that Lim et al. (2013) were the only ones focused on a cognitively intact older adult population. However, that study was longitudinal, with a higher sample size than ours (*N* = 165), without contemplating sex differences and where Aβ levels were also taken into account. Thus, we believe that none of the cited references challenge our results. Secondly, and regarding the analytical procedure, another possible limitation, particularly affecting low frequency bands results, would be the selection of 4 s length epochs. Longer epochs would increase the frequency-associated reliability, but we decided to keep 4 s, based on previous studies of our group and the good trade-off between number of clean epochs and frequency resolution (Nakamura et al., 2017; Dimitriadis et al., 2018). Thirdly, we are aware that the FC metric (PLV) could be theoretically affected by source leakage. However, we believe that this is not the case for our results since: (a) the analysis conducted consisted on a between-groups comparison. This approach reduces the possibility of having false results driven by source leakage as this problem would be affecting both groups equally and (b) the subnetwork described in this paper was composed by long distance links, which are less likely to be affected by this issue.

## Conclusions

This study demonstrates that multimodal analyses, combining MEG FC and genetics, offer valuable information in the assessment of possible selective brain network vulnerabilities associated with the beginning of the complex continuum from healthy to pathological aging. Furthermore, the non-invasiveness of MEG may be also useful as a surrogate endpoint for monitoring the effects of future pharmacological and non-pharmacological treatment interventions.

## Author contributions

ICR-R, PC, and ML designed the final study. PM, MM-P, AM, and RL-H were in charge of the initial selection of subjects and the neuropsychological part of the manuscript. AB performed the genetic determinations. PC, ICR-R, ML, JdF-L, RB, EP, AF, and FM contributed to the MEG analyses and interpretation of data. ICR-R and PC drafted the manuscript and all authors revised and approved the final version.

### Conflict of interest statement

The authors declare that the research was conducted in the absence of any commercial or financial relationships that could be construed as a potential conflict of interest. The handling Editor declared a shared affiliation, though no other collaboration, with one of the authors JdF-L.
